# Why should mental health have a place in the post-2015 global health agenda?

**DOI:** 10.1186/1752-4458-8-38

**Published:** 2014-10-11

**Authors:** Peter McGovern

**Affiliations:** Health Improvement Project Zanzibar, Makunduchi Cottage Hospital, South District Unguja, Zanzibar, Tanzania

## Abstract

**Background:**

The tenure of the Millennium Development Goals formally expires in 2015 and will be replaced with a new development agenda. The MDGs did not include goals or targets for mental health. Despite gathering momentum during the last 15 years, mental health has not enjoyed the same pace of progress as the sectors explicitly mentioned within the MDGs. This article outlines the evidence indicating that mental health should be firmly positioned in post-2015 health policy and discusses strategies to advance the global mental health agenda.

**Discussion:**

The interactions between mental health and other development goals are numerous and complex. Consequently, investment in mental health pays dividends on a wider level than simply psychiatric clinical outcomes. Mental health’s reciprocal relationship with poverty is consistent with the strong focus on economic development, rather than health in isolation, detailed in the post-2015 UN statements to date. A focus on the quality of mental health care provided in low and middle-income countries deserves priority in the new health agenda. This should include consideration of the accessibility of mental health care and the use of evidence based diagnosis and management in these settings.

**Summary:**

Lack of investment in the mental health of populations is a key driver of poverty and inequality in low and middle-income countries. Renewed focus on mental health post-2015 is an opportunity to address the global burden of mental disorders and make a positive impact on the wider development agenda.

In the year 2000, the Millennium Declaration and subsequent Millennium Development Goals (MDGs) set out the next generation of global development policy. These goals not only focused the world’s attention on the challenges of global development, but gave centre stage to a group of health challenges facing the world’s poorest. This international agenda, whose tenure formally expires next year, has guided health budgets of national governments, NGO’s and the wider aid community for 15 years. As the world reflects on their successes and failures it also seeks to choreograph the post-2015 development agenda. Despite accounting for 13% of the global burden of disease, of which low and middle-income countries hold 70%, neuropsychiatric disorders, did not feature in the previous MDGs [1]. By 2020 an estimated 1.5 million people will die each year from suicide and the number of people making an attempt will increase to between 15 and 30 million [2].

The 2013 UN report “A New Global Partnership”, a vision statement from experts on the post-2015 development agenda makes it clear that there will be renewed focus on eradication of poverty and sustainable consumption and development [3]. There is a clear appetite to frame the world’s development agenda within the context of climate change and unite the environmental agenda with development. This new agenda is broader in nature and is focused on transformative themes with a stronger focus on economic development rather than health in isolation. The economic argument for mental health investment is well documented. It is estimated that over the next 20 years mental health disorders will amount to a US$16 trillion loss of economic output [4].

The first draft of the “Overarching Health Goal” [5] produced by the WHO, (unveiled at the 2014 World Health Assembly) will form the basis of the UN post-2015 health agenda and has included mental health. Sub-goal 2, which deals with non-communicable diseases and mental illness, includes a provision to “reduce deaths and disabilities from injuries and mental disorders”. As yet this sub-goal does not have any specific targets for 2030 but it seems current momentum may see mental health having a place in the development agenda.

## Have the MDG’s affected the global health agenda?

The inextricable focus on health within the MDGs was designed to coordinate global actors working in health development. Shared goals, targeted at specific aims, were designed to achieve better and more coordinated results. They did not however constitute a radical departure in global focus.

Manning points out that “health and in particular child health has long been a natural focus for the donor community and development advocates” [6]. The focus on maternal health for instance dates back to 1985 and a provocative article “Where is the M in MCH” [7].

The MDGs have however had an effect on funding. Donor organisations like the Global Fund were born out of the MDG agenda and have focused over $23 billion dollars of aid on 3 conditions since 2002 [8]. These disease-specific interventions are regarded as reflective of the close coherence of donor policy and the MDGs over the last 15 years [2]. This raises the question what does this mean for the large cohort of global issues that have not been included, in particular what effect have the MDGs had on the “almost entirely ignored non-communicable diseases including mental health disorders”? [9].

## Mental health as a priority in the developing world

The successes in global health over the last 15 years have not included gains for mental health in lower resource settings. Between 76% and 85% of people with severe mental disorders receive no treatment for their condition in low-income and middle-income countries [10]. Worse still, this treatment gap fails to account for the low standards of care provided for those that do receive treatment. Chronic underfunding and lack of investment in services have rendered mental health programmes struggling to function and unable to attract funding. Mean annual spending on mental health services per capita in low-income countries is only US$0.25 [9] and this paltry funding is primarily targeted at larger inpatient services traditionally associated with poor outcomes and abuses of human rights. The mental health sector has gathered some momentum during the tenure of the Millennium Development Goals, including the release of the WHO mental health gap action programme and the Lancet Series on global mental health [11], but not at the same pace as those sectors explicitly mentioned within the MDGs.

The mental health community has however continued to show that investment in mental health services pays dividends on a wider level than simply psychiatric clinical outcomes. People with mental disorders continue to have increased rates of disability and premature mortality. As an example, WHO has reported that persons with major depression or schizophrenia have a 40-60% greater chance of dying prematurely compared with the general population [10]. Much of this increased risk is due to comorbid physical illness that goes untreated. Targeting mental health of populations can help to address specific aims within the current MDGs and reflect the goals of even disease specific donors. A 2005 paper makes the case that tackling mental health problems should be viewed as an integral part of health system interventions and that mental health has direct implications for the attainment of the MDGs [9]. As an example, one of the barriers to universal primary education, MDG 2, is a lack of inclusive education. Children with mental disorders or learning disabilities are frequently excluded from primary education [12]. A focus on mental health services for these children, early diagnosis and liaison between healthcare and education staff could improve education enrolment and completion. With regard to MDG 6, there is an association of depression with disease progression in HIV/AIDS and mental disorders have been shown to impede adherence to antiretroviral therapy [13]. Another example in Peru showed that group psychotherapy could improve adherence to treatment for patients with multi-drug resistant Tuberculosis [14].

Mental disorders also have a reciprocal relationship with poverty. An overwhelming majority of people with mental disorders live in poverty, with population-based studies of risk factors for mental disorder showing that poor and marginalised people are at a greater risk of suffering from these conditions [15]. Mental disorders themselves can lead individuals and their families into poverty due to the cost of treatment, often availed of through the private sector, and also results in lost employment opportunities [9]. The relationship is complex yet undeniable, and action on mental health could prevent this negative poverty cycle.

A common argument for the lack of focus on mental health in the international development agenda is that in poverty, the more urgent issues are the serious physical health problems facing populations. In response, however, the case has been convincingly made that “there is no health without mental health” [16]. The interactions of physical and mental health are numerous and complex, and necessitate explicit recognition in health system development. A holistic approach that takes into account the links between physical and mental health, as seen in certain high-resource settings, must reach those who historically have been unable to access comprehensive physical and mental health care.

## What can be done to advance the mental health agenda?

Any approach to prioritising mental health in the development agenda should seek to address the many systemic barriers that prevent improvements in the mental health of populations. For many living in low and middle-income countries mental health care is simply not available. When it is available one clear barrier is poor quality care. Shidhaye points out that only 10% of people receive any evidence-based treatment, even when they do overcome the barriers of access and attend for treatment [17]. This lack of evidence-based treatment in mental health around the world is leading to under-detection, and often total lack of detection of mental health disorders, further widening the treatment gap. A systemic lack of resources, inequity in resource distribution and inefficiency in mental health systems further entrenches poor quality care [18] as well as its overall unavailability.

Mental health resources remain stubbornly centralised without the required development of community mental health services that could fuel population wide progress [19]. Stigma toward mental health further constrains how these scarce resources are used and can leave people with mental disorders vulnerable to human rights abuses [18]. This focus on a human rights based approach to healthcare was not translated from the Millennium Declaration to the MDGs. Nor did the MDGs focus on how low quality services, and the presence of systemic weaknesses in health infrastructure presents barriers to healthcare improvements.

The lack of quality in the current care provided, demonstrates the need for more holistic means of measuring the targets set in the post 2015 agenda. Targets should address not only the provision of care but the quality of the care provided. The current draft of the WHO “Overarching Health Goal” calls for 80% coverage of services in all population groups, including severe mental disorders by 2030, as part of sub goal 3 “Achieve Universal Health Coverage” [5]. This demonstrates a willingness to address the availability of mental health care however, in its current form, this health goal remains silent on the issue of quality.

Farooq [20] suggests that answers lie in an integrated model of care for mental health that is collaborative with other non-communicable diseases. He suggests this is a cost effective as well as successful way of providing care [20]. It is possible that this style of integration with physical health will draw attention and funding to those programmes dealing with mental health. This is an example of innovative solutions that the mental health community are progressing on to achieve the vision set out by the WHO mental health action plan “a world in which mental health is valued, promoted and protected” [10].

If we are to realise this vision then mental health needs to play an integral and integrated role in the post 2015 agenda [21]. National governments that have benefited from their investment in mental health, to help achieve MDG targets, have the opportunity to show leadership and lobby for its inclusion in the future development agenda. The wider mental health community must press the UN and influential development organisations for a radical new focus on mental health post-2015. We must be clear that this investment is a means by which to galvanise the development successes over the last 15 years as well a driver for new progress that will benefit the most vulnerable populations. This disease burden is held by the worlds poorest and compounds their experiences of poverty and often perpetuates abuses of their human rights. Lack of investment in the mental health of populations is a key driver of poverty and inequality in low-income countries. The post 2015 agenda to eradicate poverty and drive sustainable development will only have its desired transformative effect if the mental health of the world, especially in its most vulnerable populations, is given due attention.

## References

[1] Lopez DA, Mathers DC, Ezzati M, Jamison DT, Murray CJ (2006). Global Burden Of Disease And Risk Factors.

[2] Collins P, Patel V (2011). Grand Challenges in global mental health. Nature.

[3] United Nations (2013). A New Global Partnership: Eradicate Poverty And Transform Economies Through Sustainable Development. The Report Of The High-Level Panel Of Eminent Persons On The Post 2015 Development Agenda.

[4] Bloom DE, Cafiero ET, Jané-Llopis E, Abrahams-Gessel S, Bloom LR, Fathima S, Feigl AB, Gaziano T, Mowafi M, Pandya A, Prettner K, Rosenberg L, Seligman B, Stein AZ, Weinstein C (2011). The Global Economic Burden Of Noncommunicable Diseases.

[5] World Health Organisation (2014). Overarching Health Goal (Draft): Ensure Healthy Lives And Universal Health Coverage At All Ages.

[6] Manning R (2010). The Impact And Design of the MDG’s: Some Reflections. IDS Bulletin Volume 41 Jan 2010.

[7] Gruskin S, Cottingham J (2008). Using Human rights to improve maternal and neonatal health. History, connections and a proposed practical approach. Bull World Health Org.

[8] The Global Fund 2014: *Annual Disbursements Summary 2002–2013*. [http://www.theglobalfund.org/en/about/fundingspending/#disbursed]

[9] Miranda J, Patel V (2005). Achieving the Millennium Development Goals: Does Mental Health Play a Role?. PLoS Med.

[10] World Health Organisation 2013 (2013). Mental Health Action Plan 2013–2020.

[11] Patel V, Garrison P, de Jesus Mari J, Minas H, Prince M (2008). The Lancet’s Series on Global Mental Health: 1 year on. Lancet.

[12] Patel V, De Souza N (2000). School drop-out: A public health approach for India. Natl Med J India.

[13] Freeman M (2005). Integrating mental health in global initiatives for HIV/AIDS. British J Psychiatry.

[14] Sweetland A, Albujar JA, Echevarria DG (2002). Enhancing Adherence: The Role Of Group Psychotherapy In The Treatment of MDR-TB in Urban Peru. In World Mental Health Casebook: Social and Mental Health Programmes in Low-income Countries.

[15] Patel V, Kleinman A (2003). Poverty and common mental disorders in developing countries. Bull World Health Org.

[16] Prince M, Patel V, Saxena S, Maj M, Maselko J, Phillips M, Rahman A (2007). No health without mental health. Lancet.

[17] Shidhaye R, Kermode M (2013). Stigma and discrimination as a barrier to mental health service utilization in India. Int Health.

[18] Saxena S, Thornicroft G, Knapp M, Whiteford H (2007). Resources for mental health: scarcity, inequity and inefficiency. Lancet.

[19] Saraceno B, Van Ommeren M, Batniji R, Cohen A, Gureje O, Mahoney J, Sridhar D, Underhill C (2007). Barriers to improvement of mental health services in low-income and middle-income countries. Lancet.

[20] Farooq S (2013). Collaborative care for depression: a literature review and a model for implementation in developing countries. Int Health.

[21] Demyttenaere K, Bruffaerts R, Posada-Villa J, Gasquet V, Kovess JP, Lepine MC, Angermeyer S, Bernert G, De Girolamo P, Morosini G, Polidori T, Kikkawa N, Kawakami Y, Ono T, Takeshima H, Uda EG, Karam JA, Fayyad AN, Karam ZN, Mneimneh ME, Medina-Mora G, Borges C, Lara R, De Graaf J, Ormel O, Gureje YC, Shen YQ, Huang MY, Zhang J, Alonso JM (2004). Prevalence, severity, and unmet need for treatment of mental disorders in the World Health Organization World Mental Health Surveys. JAMA.

